# A Deadly Complication of Superficial Muscular Needle Electromyography: Bilateral Pneumothoraces

**DOI:** 10.1155/2013/861787

**Published:** 2013-12-10

**Authors:** Erden Erol Ünlüer, Pınar Yeşim Akyol, Arif Karagöz, Serkan Bılgın

**Affiliations:** Emergency Department, Izmir Katip Celebi University, Ataturk Research and Training Hospital, 35360 Izmir, Turkey

## Abstract

Needle electromyography (EMG) is an expression of the physiological or pathophysiological state of muscles. Selection of the type of electrode used during these measurements is based upon several factors, including the location of the muscle of interest, the need for specificity, and the requirement of minimization of cross-talk between adjacent muscles. Pneumothorax is a serious complication of needle EMG. Here, we present a 19-year-old patient who suffered bilateral pneumothoraces as a complication of needle EMG. She has a history of weakness and limitation of abduction on her right shoulder for three years. EMG was ordered by orthopedic surgeon to determine whether a dorsal scapular or long thoracic nerve paralysis caused these symptoms. She was brought to our emergency department (ED) with the complaints of diaphoresis and dyspnea which began after needle EMG was performed two hours ago. A chest X-ray revealed bilateral small pneumothoraces and was confirmed by computed thoracic tomography scan. Patient was admitted to observation unit in ED. Thoracic ultrasonography was preferred to follow up the patient. After five days, pneumothoraces were dissolved on bilaterally and the patient was discharged to home. Iatrogenic pneumothorax is a complication observed at various clinical fields. Emergency physician must consider this possibility in patients admitted with dyspnea after needle EMG.

## 1. Introduction

Needle electromyography (EMG) is an expression of the physiological or pathophysiological state of muscles. The electrical potentials are observed after the needle electrode is inserted into the muscle. In patients with assumed peripheral nerve damage EMG is designated to differentiate damage located in muscle or nerve; if damage is present, it can be used to measure severity of damage. It is useful in pinpointing the exact location of damage [1]. Selection of the type of electrode used during these measurements is based upon several factors, including the location of the muscle of interest, the need for specificity, and the requirement of minimization of cross-talk between adjacent muscles. Deep muscles require the use of fine wire electrodes, while superficial muscle activity can be detected with surface electrodes [2]. Pneumothorax is a serious complication of needle EMG [3]. Here, we present a 19-year-old patient who suffered bilateral pneumothoraces as a complication of needle EMG and to our best knowledge this was the first case of bilateral iatrogenic pneumothoraces seen as a complication of superficial muscular needle EMG.

## 2. Case

A 19-year-old girl was brought to our emergency department (ED) with the complaints of diaphoresis and dyspnea which began after bilateral needle EMG was performed two hours ago with 38 × 0.45 mm needles. She had a history of weakness and limitation of abduction on her right shoulder for three years. EMG was ordered by orthopedic surgeon to determine whether a dorsal scapular or long thoracic nerve paralysis caused these symptoms. Her temperature was 36.6°C, blood pressure was 90/60 mmHg, pulse rate was 106/min, respiratory rate was 24/min, and oxygen saturation was 96% with pulse oximeter. On physical examination, breath sounds were decreased on both sides of thorax; the rest of the examination was normal. Her past medical history was unremarkable.

**Figure 1:**
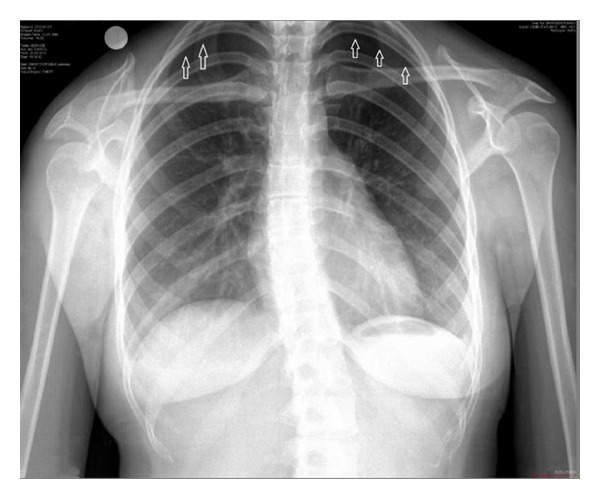
Chest X-ray shows bilateral small pneumothoraces. White arrows indicate pleural lines.

**Figure 2:**
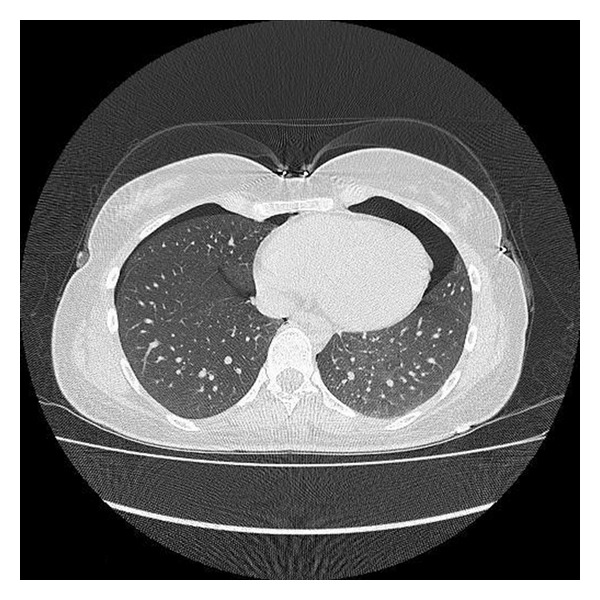
Computed tomography scan shows bilateral pneumothoraces.

Initial investigations on admission confirmed normal hemogram, routine blood chemicals, and blood gas analysis. A chest X-ray (CXR) revealed bilateral small pneumothoraces and was confirmed by computed thoracic tomography (CT) scan (Figures 1 and 2). Patient was admitted to observation unit in ED. Analgesic therapy when needed together with oxygen therapy was started with 100% oxygen from a nonrebreathing reservoir mask. Thoracic ultrasonography was preferred to follow up the patient to see the pleural sliding movements and comet tail artifacts synchronized with the patient breathing. After five days, pneumothoraces were dissolved on bilaterally and the patient was discharged to home.

## 3. Conclusion

Pneumothorax is defined as the presence of air in the pleural space [4]. Iatrogenic pneumothorax refers to the pneumothorax generated after diagnostic or treatment procedure and is a complication observed at various clinical fields. Recently, the incidence of iatrogenic pneumothorax is increased with the development of invasive procedures for various diagnostic or treatment purposes [5]. Simultaneous bilateral pneumothorax is a rare clinical event, the causes of which include trauma, tumor, tuberculosis, and iatrogenic situations arising from central line placement or intubation [6].

The diagnosis should be based on clinical and radiological findings. On physical examination, there is reduced breast wall movement, hyperresonance, and decreased or lost respiratory sounds. The patient may also have tachycardia, subcutaneous emphysema, and dyspnea. If there is tension pneumothorax, shock may develop [7]. Our patient had no tension pneumothorax because she had normal vital signs and had not tachycardia. Additionally, on CXR, there was no mediastinal shift and pneumothoraces were less than 2 cm on both sides.

The standard diagnostic method for the detection of pneumothorax is CT, but CXR is commonly used as a first line diagnostic test. However, CT and CXR can require time to perform and carry the risk of radiation exposure. Ultrasound is more easily available in pain management as a diagnostic and practical tool. Ultrasound examination of the chest is simple, economical, and free of radiation, and ultrasound has been described as a more accurate method than CXR for the detection of pneumothorax [8, 9].

Managements of pneumothorax can include observation, simple aspiration, or tube thoracotomy, depending on the size of the pneumothorax, symptoms, presence of continued air leak, and evidence of a tension pneumothorax [10]. The absorption rate of the air is about 1.25% (50–75 mL)/day. However, if pneumothorax increases during observation, chest tube drainage may be required [6, 7].

Emergency physician must consider the possibility of iatrogenic pneumothorax in patients admitted with dyspnea after needle EMG.
